# Rho GTPases in cancer resistance: mechanisms, vulnerabilities, and therapeutic opportunities

**DOI:** 10.1042/BST20250097

**Published:** 2026-05-07

**Authors:** Amy Beaudin, Philippe Lefrançois, Mélanie Laurin

**Affiliations:** 1 Département de biologie moléculaire, biochimie médicale et pathologie, Faculté de médecine, Université Laval, Québec, QC, Canada; 2 Oncology Research program, CHU de Québec - Université Laval Research Center, Québec, QC, Canada; 3 Centre de recherche sur le cancer de l'Université Laval (CRC), Québec, QC, Canada; 4 Centre de recherche en organogénèse expérimentale (LOEX), Québec, QC, Canada; 5 Cancer Axis, Lady Davis Institute for Medical Research, Montreal, QC, Canada; 6 Division of Experimental Medicine, McGill University, Montreal, QC, Canada; 7 Division of Dermatology, Department of Medicine, McGill University, Montreal, QC, Canada

**Keywords:** cancer, chemotherapy resistance, Rho GTPase, signalling, small molecules, treatment resistance

## Abstract

Intrinsic and adaptive resistance to therapy remain major barriers to effective cancer treatment. Diverse resistance mechanisms, including epithelial-mesenchymal transition, enhanced tolerance to DNA damage, impaired cell death pathways, metabolic reprogramming, and cues from the tumour microenvironment, are increasingly recognised as being tightly integrated with Rho GTPase signalling networks. Accumulating evidence positions these pathways as central regulators of therapeutic resistance across multiple cancer types. In this review, we synthesise recent experimental findings linking Rho GTPase-mediated signalling to therapy resistance and evaluate emerging strategies aimed at targeting these signalling axes. We critically examine the translational readiness of approaches that directly inhibit Rho GTPases, disrupt downstream effector pathways, or modulate canonical regulators such as RhoGEFs and RhoGAPs, and discuss the key challenges and opportunities associated with their clinical deployment. Collectively, these insights highlight the therapeutic potential of targeting Rho GTPase signalling as a foundation for next-generation cancer treatments.

## Rho GTPases in cancer biology

The Rho family of small GTPases functions as a central signalling hub integrating extracellular cues to coordinate cytoskeletal dynamics, transcriptional programmes, and cellular stress responses [1]. In humans, this family comprises 20 members, with RAC1, RHOA, and CDC42 being the most extensively studied [2,3]. Although oncogenic mutations in Rho GTPases have been identified, these events are rare relative to Ras mutations, and Rho GTPase-driven oncogenesis more commonly reflects oncogene-driven or therapy-induced signalling rewiring rather than direct genetic lesions [4–13]. Oncogenic inputs from receptor tyrosine kinases, G protein-coupled receptors, and integrins converge on Rho GTPase regulatory networks. Within these, guanine nucleotide exchange factors (GEFs) promote GDP–GTP exchange and activation, whereas GTPase-activating proteins (GAPs) terminate signalling by accelerating GTP hydrolysis [4,14–16]. Disruption of this regulatory balance promotes tumour initiation, progression, and therapeutic resistance by altering proliferation, survival, migration, genome integrity, and stress adaptation [17]. This rewiring enables tumour cells to bypass drug-inhibited nodes through activation of parallel signalling pathways, reinforcement of pro-survival and DNA damage-repair programmes, and enhanced tolerance to genotoxic and metabolic stress. Collectively, Rho GTPases act as integrators of metastatic potential and therapy failure, reinforcing their value as therapeutic targets.

## RAC1-dependent signalling in multimodal therapy resistance

**Figure 1:**
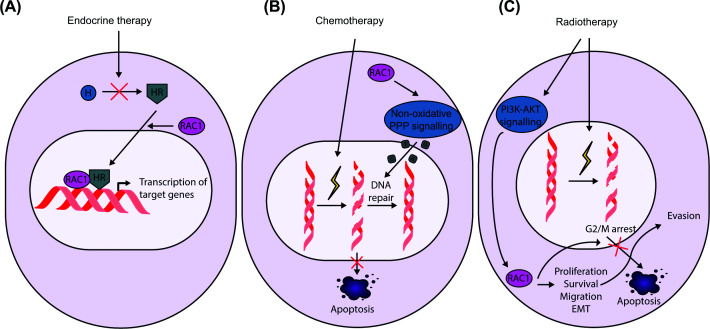
RAC1 signalling contributes to resistance across diverse cancer therapies (**A**) RAC1 drives endocrine therapy resistance by sustaining hormone receptor stability, chromatin association and hormone-dependent transcriptional programmes. (**B**) RAC1 promotes resistance to neoadjuvant chemotherapy through metabolic rewiring that enhances nucleotide biosynthesis and supports DNA repair. (**C**) Elevated RAC1 expression drives radiotherapy resistance by promoting proliferation, epithelial-to-mesenchymal transition EMT, and G2/M checkpoint activation downstream of PI3K-AKT signalling.

RAC1 has emerged as a central regulator of adaptive resistance across multiple therapeutic modalities [18]. In breast cancer, RAC1 integrates hormone signalling, growth factor pathways, and metabolic rewiring to promote treatment failure. A key mechanism involves RAC1-dependent regulation of estrogen receptor (ER) protein stability and transcriptional activity. ER-positive breast cancer cell lines require RAC1 for proliferation and are more sensitive to RAC1 perturbation than ER-negative counterparts. Mechanistically, RAC1 depletion or pharmacological inhibition via EHT1864 induces ER degradation and suppresses ER-dependent transcription, partly by impairing RNA polymerase II activity, even in the presence of estrogen (Figure 1A and Table 1) [19,20]. RAC1 plays a similar role in prostate cancer, where it sustains androgen receptor (AR)-dependent transcription (Figure 1A). In prostate cancer cell models, pharmacological inhibition of RAC1 via GYS32661 suppresses proliferation and survival of androgen-dependent cells while potentiating AR antagonists and their effects on AR target gene expression, supporting a conserved requirement for RAC1 in hormone-driven transcriptional programmes (Table 1) [21,22].

**Table 1:** Overview of inhibitors targeting Rho GTPase-mediated signalling

Inhibitors	Specificity	Mechanism of action	Ref
**Direct Rho GTPase inhibitors**			
ML141	CDC42	Inhibition of Rho GTPase activity by binding allosteric cavity.	[94]
EHT 1864	RAC1	Inhibition of Rho GTPase activity by binding allosteric cavity.	[94]
R-ketorolac	CDC42, RAC1	Interferes with the intrinsic GTPase activity by binding near switch I	[94]
Compound 7	CDC42, RAC1, RHOA	Inhibition of Rho GTPase activity by binding allosteric cavity.	[109]
JK-136	RHOA	Inhibits Rho GTPase activity by binding active site	[110]
GYS32661	RAC1	Inhibits nucleotide binding	[111]
Rhosin	RHOA	Directly binds RHOA to prevent its activation	[112]
**GEF interaction inhibitors**			
A13	RHOA	Blocks interaction with GEF LBC.	[113]
MBQ-167	CDC42, RAC1	Binds the switch I region and inhibits GEF binding.	[114]
EHop-016	CDC42, RAC1	Blocks interaction with GEF VAV2.	[115]
1A-116	RAC1	Inhibits RAC1–PREX1 interaction	[116]
NSC23766	RAC1	Blocks interaction with GEF TIAM1	[117]
Peptide FGDWS	RAC1	Inhibits RAC1–TIAM1 interaction	[118]
ZINC69391	RAC1	Inhibits RAC1–TIAM1 and RAC1–DOCK1 interactions	[116]
HV-107	RAC1	Blocks interaction with GEF VAV2.	[119]
ZCL278	CDC42	Inhibits CDC42–ITSN interaction	[120]
AZA197	CDC42	Inhibits interactions with DBL GEFs.	[121]
ZCL367	CDC42	Inhibits CDC42–ITSN interaction	[122]
CASIN	CDC42	Inhibits CDC42–ITSN interaction	[123,124]
TBOPP	RAC1	Inhibits RAC1–DOCK1 interaction	[125]
IODVA1	RAC1	Blocks interaction with GEF VAV3	[126,127]
Y16	RHOA	Inhibits RHOA–LARG (ARHGEF12) interaction	[128]
**Downstream effectors inhibitors**			
Frax597	RAC1, CDC42	Inhibition of PAK	[129]
Frax-1036	RAC1	Inhibition of PAK	[130]
H1152	RHOA	Inhibits ROCK1/ROCK2	[131]
RKI-1447	RHOA	Inhibits ROCK1/ROCK2	[132]
AT13148	RHOA	Inhibits ROCK1/ROCK2	[133]
ARN22089 ARN25062 ARN24928	CDC42	Inhibition of PAK	[134–136]
Y-27632	RHOA	Inhibits ROCK1/ROCK2	[137]

While not exhaustive, this table groups the most common inhibitors based on their mechanism of action described in the text.

Beyond sex hormone-dependent carcinogenesis, RAC1 also contributes to resistance to tyrosine kinase inhibitors, particularly EGFR- and HER2-targeted therapies. Gefitinib- and lapatinib-resistant breast cancer cell lines exhibit elevated RAC1 expression and activity, and pharmacological inhibition of RAC1 with EHop-016 or dual inhibition of RAC1/CDC42 with MBQ-167 restores sensitivity to either agent (Table 1) [23]. Hyperactivation of RAC1 similarly promotes melanoma cell growth and resistance to BRAF inhibitors, although this effect appears restricted to a dedifferentiated melanoma subtype in which RAC1 maintains cellular dedifferentiation [24]. However, further studies are required to define the resistance-enabling molecular mechanisms engaged in these contexts.

RAC1 also promotes resistance to chemotherapy and radiotherapy. The majority of studies support a model in which RAC1 promotes survival under genotoxic stress by facilitating checkpoint activation and DNA repair. In breast cancer, RAC1 promotes resistance by rewiring cell metabolism through aldolase A- and ERK-dependent metabolic pathways. This reprogramming enhances glycolytic flux through the non-oxidative pentose phosphate pathway, increasing nucleotide biosynthesis to support DNA repair and survival under genotoxic stress (Figure 1B) [25]. Notably, systemic delivery of *RAC1*-targeting siRNA via nanoparticle-based approaches restores chemosensitivity in patient-derived xenograft (PDX) models, highlighting the therapeutic potential of targeting RAC1 [25]. Similarly, in haematological malignancies, RAC1 can promote chemoresistance through interaction with TNFAIP8, enhancing ERK signalling and suppressing apoptosis [29]. In this context, RAC1 inhibition with EHOP-016 reduces ERK activation even in TNFAIP8-overexpressing cells (Table 1). Whether RAC1 inhibition alone phenocopies TNFAIP8 loss or sensitises AML cells to chemotherapy remains unresolved. RAC1 also contributes to chemoresistance in breast cancer through an integrin β4/TNFAIP2/IQGAP1/RAC1 axis that supports DNA repair [26]. Indeed, knockdown of components of this axis restores the sensitivity of the cells to chemotherapeutic drugs. RAC1-driven adaptive signalling further extends to radiotherapy resistance. In non-small cell lung cancer cells, ionising radiation induces RAC1 up-regulation through PI3K-AKT signalling, increasing proliferation, clonogenic capacity, and mesenchymal features associated with radioresistance. Moreover, shRNA-mediated depletion sensitises cell-line-derived xenografts (CDX) to radiotherapy [27]. Consistently, RAC1-dependent radioresistance has also been reported in breast and pancreatic cancer models. In these, RAC1 mediates ionising radiation-induced G2/M arrest, and its inhibition with NSC23766 prevents activation of the DNA damage response kinases ATM and ATR, while reducing ERK signalling and promoting radiosensitisation (Figure 1C) [28].

However, while the preponderance of evidence supports a pro-repair role, RAC1 can also enhance DNA damage through distinct effector pathways. Indeed, RAC1 can activate NADPH oxidases (NOX), leading to increased production of reactive oxygen species and consequent DNA damage. For instance, Zhang et al. recently reported that ARHGAP15 promotes metastatic colonisation in gastric cancer by suppressing the RAC1–NOX2 axis, thereby protecting tumour cells from oxidative stress [29]. This mechanism may similarly influence responses to therapies that rely on oxidative stress. Importantly, in acute myeloid leukaemia, the role of RAC1 also differs from its predominantly cancer promotion and drug resistance roles. In this context, resistance to doxorubicin arises from RAC1 inhibition rather than activation. Under genotoxic stress, RAC1 interacts with topoisomerase II to promote DNA double-strand breaks and apoptosis, and RAC1 inhibition with NSC23766 reduces doxorubicin-induced DNA damage (Table 1) [30]. Thus, in AML treated with topoisomerase II poisons, RAC1 activity appears to facilitate drug-induced cytotoxicity rather than protect against it [29].

Altogether, these findings suggest that the impact of RAC1 on genomic stability depends on the balance between its roles in checkpoint activation, survival signalling, oxidant production, and modulation of DNA-processing enzymes and is highly context-dependent.

## Alternative RAC activation mechanisms: RAC1B and oncogenic RAC1 variants

RAC hyperactivation can occur independently of upstream regulatory inputs. The alternatively spliced isoform RAC1B is constitutively active and expressed across multiple cancers [22,31–35]. In colorectal cancer, RAC1B expression increases with disease progression and chemotherapy exposure. RAC1B depletion or inhibition with GYS32661 sensitises colorectal cancer cells to oxaliplatin by preventing NF-κB activation (Table 1) [22]. RAC1B is also expressed in luminal A breast cancer cells, where it is required for doxorubicin resistance and tumour-initiating capacity in CDX. Consistently, loss of RAC1B in a HER2-driven mouse model impairs tumour formation and increases chemosensitivity to doxorubicin, while elevated RAC1B levels predict poorer survival in doxorubicin-treated patients [31]. *RAC1* mutations further expand this resistance landscape. In melanoma, *RAC1^P29S^* is the third most common somatic mutation after *BRAF* and *NRAS* and encodes a fast-cycling GTPase that promotes hyperactivation of downstream signalling [36,37]. RAC1^P29S^ has been implicated in early resistance to RAF inhibitors, where it sustains MAPK signalling and drives a mesenchymal transcriptional programme via activation of the SRF/MRTF axis, thereby supporting its proposed role as a biomarker of intrinsic resistance to RAF-targeted therapies [38–41]. However, this relationship remains debated. Recent work from the Rosen laboratory demonstrated that BRAF^V600E^ induces a negative feedback loop that restrains RAC activity. Pharmacological inhibition of the ERK pathway releases this constraint, resulting in RAC activation. In this model, acquisition of *RAC1^P29S^* in BRAF-mutant tumours could permit sustained RAC signalling that bypasses feedback control, potentially contributing to tumour progression rather than directly mediating drug resistance [42]. Together, these findings highlight the context-dependent and dynamic interplay between RAC1 and MAPK signalling in the context of targeted therapy.

## Targeting RAC regulators as a therapeutic strategy

Targeting RAC regulators may provide greater therapeutic leverage than inhibiting RAC1 alone. DOCK1 was identified as a synthetic-lethal vulnerability in metformin-treated hepatocellular carcinoma [43]. Metformin promotes DOCK1 phosphorylation, RAC1 activation and enhanced cell survival. Accordingly, genetic depletion of DOCK1 or its pharmacological inhibition with TBOPP, a small-molecule inhibitor that binds DOCK1 and disrupts its interaction with RAC1 to suppress GEF activity, sensitises patient-derived organoids and mouse liver cancer models to metformin (Table 1) [44,45]. Clinically, metformin treatment through drug repurposing from its original use in diabetes is associated with improved survival in patients with low DOCK1 expression, supporting the potential use of DOCK1 as a biomarker for patient stratification [43]. Beyond liver cancer, DOCK1 inhibition also sensitises breast cancer cells to cisplatin *in vitro* and in CDX. This effect is mediated by suppression of the EMT regulator TWIST and is reversed by its enforced re-expression, establishing a functional DOCK1–TWIST axis in chemoresistance [46]. Consistent with this, DOCK1 inhibition similarly restores cisplatin sensitivity in bladder and renal cell carcinoma models [47,48].

DOCK6, a RhoGEF for RAC1 and CDC42, is overexpressed in gastric cancer and correlates with poor prognosis. Elevated DOCK6 levels are associated with activation of WNT/ß-catenin signalling and acquisition of cancer stem cell-like features, conferring resistance to chemotherapy and radiotherapy. In contrast, DOCK6 depletion restores therapeutic sensitivity, a phenotype mediated primarily through RAC1 rather than CDC42, as RAC1 knockdown in DOCK6-overexpressing cells reinstates treatment responsiveness [49]. Beyond the DOCK family, pharmacological disruption of VAV3 using the small molecule IODVA1 overcomes resistance to tyrosine kinase inhibitors and reduces leukemic burden in PDX (Table 1) [50].

These findings underscore the therapeutic potential of targeting the RAC signalling axis, including its upstream activators, in drug-resistant cancers. While supported by robust mechanistic and preclinical *in vivo* evidence, translational advancement remains constrained by tumour-context dependency and an incomplete understanding of isoform-specific and mutant RAC functions. Future efforts should focus on developing selective targeting strategies and implementing biomarker-guided clinical validation to determine whether RAC-directed therapies can effectively overcome resistance in patients.

## CDC42-driven mechanisms of collateral and acquired chemotherapy resistance

**Figure 2:**
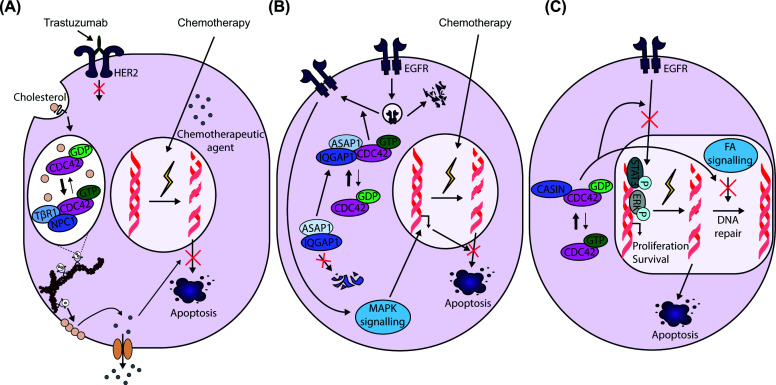
CDC42-mediated mechanisms of collateral and acquired chemotherapy resistance (**A**) Collateral resistance to chemotherapy in trastuzumab-resistant HER2-positive cancers is mediated by endosomal activation of CDC42, which promotes cholesterol trafficking-dependent pro-survival signalling and drug tolerance. (**B**) CDC42 promotes chemotherapy resistance through an ASAP1–IQGAP1 axis that amplifies EGFR–MAPK signalling and contributes to reduced drug sensitivity. (**C**) Inhibition of CDC42 by CASIN restores sensitivity to melphalan and bortezomib by enhancing DNA damage and suppressing EGFR–STAT3 signalling, respectively.

Collateral resistance, defined as resistance to a second therapy without prior exposure, represents a major barrier to effective cancer treatment. This phenomenon is particularly relevant in advanced HER2-positive gastric cancer, where acquired resistance to trastuzumab is often accompanied by reduced sensitivity to conventional chemotherapies. Recent mechanistic studies identify CDC42 as a key mediator of this collateral resistance. In trastuzumab-resistant cells, CDC42 is selectively activated within endosomal compartments through association with TGFβR1 and NPC1. This endosome-localised signalling axis enhances intracellular cholesterol trafficking, leading to plasma membrane cholesterol enrichment that sustains pro-survival signalling and promotes drug tolerance (Figure 2A). Pharmacological inhibition of CDC42 via ZCL278 reduces plasma-membrane cholesterol levels and reverses collateral resistance in mouse models (Table 1) [51]. Consistent with this mechanism, dual RAC1/CDC42 inhibition using MBQ-167 restores trastuzumab sensitivity in breast cancer models; whether this strategy is effective in gastric cancer remains to be established (Table 1) [52].

Beyond trastuzumab resistance, CDC42 also contributes to chemotherapy resistance in gastric cancer, including resistance to oxaliplatin. Transcriptomic analyses of cytoskeleton-associated genes identified *ASAP1* as up-regulated in gastric cancer tissues and associated with poor prognosis [53,54]. Mechanistically, ASAP1 potentiates CDC42 signalling by preventing ubiquitin-mediated degradation of the scaffold protein IQGAP1, stabilising CDC42 in its active GTP-bound state and amplifying downstream EGFR–MAPK signalling (Figure 2B) [53,55]. This ASAP1–IQGAP1–CDC42 axis drives oxaliplatin resistance in gastric tumour cells with high ASAP1 expression, and CDC42 inhibition with ZCL278 abolishes this resistance *in vitro* (Table 1) [53]. CDC42-dependent amplification of EGFR signalling has also been reported in other contexts, including castration-resistant prostate cancer cells, where CDC42 protects EGFR from degradation [56].

CDC42-dependent resistance mechanisms also extend to haematological malignancies. In multiple myeloma, resistance to melphalan and bortezomib remains a major clinical challenge. Inhibition of CDC42 with CASIN, which binds the GDP-bound form of CDC42 and prevents its activation, preferentially suppresses proliferation and survival of drug-resistant multiple myeloma cells compared with their drug-sensitive counterparts (Table 1) [57,58]. CASIN sensitises multiple myeloma cells by increasing DNA damage in melphalan-resistant cells while suppressing EGFR–STAT3 signalling and ERK activation in bortezomib-resistant cells (Figure 2C) [58].

CDC42-dependent resistance also involves extracellular matrix-derived signals. In colorectal cancer cells, fibronectin-mediated integrin ß1 activation engages a CDC42–YAP–SOX2 network that promotes resistance to 5-fluorouacil (5-FU) and cisplatin. Disruption of this pathway by integrin β1 blockade or YAP depletion restores chemosensitivity. Combining the integrin inhibitor GLPG0187 with 5-FU significantly prolongs CDX survival compared with single-agent treatments, supporting this mechanism *in vivo* [59].

Together, these findings underscore the therapeutic potential of targeting CDC42 to overcome chemotherapy resistance. Nonetheless, further validation in clinically relevant *in vivo* models is essential, given that much of the supporting evidence currently derives from cell line-based studies.

## RHOJ drives EMT-associated and DNA damage-tolerance mechanisms of chemotherapy resistance

Metastatic progression and therapeutic resistance are tightly linked to EMT in many cancers, and several Rho GTPases coordinate these processes [60]. Among them, the CDC42-related GTPase RHOJ has emerged as a regulator of EMT and tumour aggressiveness across several cancer types [61]. In gastric cancer, RHOJ is enriched in EMT-dominant subtypes and promotes invasion via IL-6-dependent STAT3 activation [62]. In clear cell renal cell carcinoma, elevated *RHOJ* expression correlates with increased EMT markers and poor outcomes, driving proliferation and invasion through TNF-α up-regulation and NF-κB activation [63]. In glioblastoma multiforme, RHOJ is transcriptionally regulated by c-Jun and promotes EMT, cell migration, and tumour growth through interaction with moesin and activation of RAC1–PAK signalling [64]. Similarly, high *RHOJ* expression in urothelial carcinoma is associated with tumour progression, impaired anti-tumour immune responses, and reduced survival [65].

**Figure 3:**
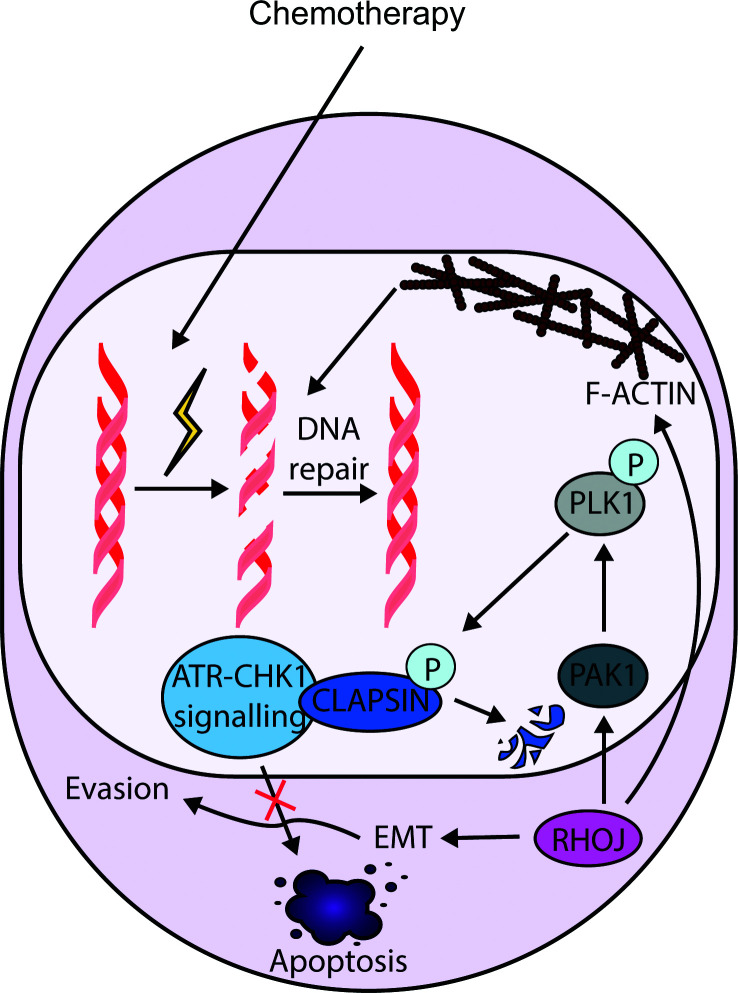
RHOJ-driven EMT and chemotherapy resistance RHOJ signalling promotes DNA damage tolerance and resistance by attenuating ATR–CHK1-dependent apoptosis and regulating nuclear cytoskeletal remodelling to support DNA repair. Elevated RHOJ is also associated with increased EMT markers expression, reinforcing tumour cell survival.

Given the link between EMT and chemotherapy resistance, RHOJ has emerged as an integrator of EMT programmes with DNA damage tolerance. Using an unbiased genome-wide RNAi synthetic-lethality screen, Ho *et al.* identified *RHOJ* and its effector PAK as key determinants of melanoma resistance to DNA-damaging agents. Following genotoxic stress, RHOJ-dependent PAK1 activation promotes PLK1-mediated degradation of the replication checkpoint protein claspin, attenuating ATR–CHK1 signalling and suppressing apoptosis. In parallel, impaired ATR signalling increases SOX10 expression, further elevating the apoptotic threshold (Figure 3) [66]. Consistent with this model, RHOJ expression is higher in melanoma cells than in melanocytes, conferring enhanced resistance to apoptosis [67]. This RHOJ–PAK survival circuit likely represents an exaggerated form of the physiological UV damage response in melanocytes.

These mechanistic insights have informed therapeutic strategies targeting RHOJ signalling. Inhibition of RHOJ-mediated signalling using the group I-selective PAK inhibitor FRAX597 has shown efficacy in melanoma models (Table 1) [68,69]. RHOJ expression is higher in BRAF^V600E^-mutant melanomas than in BRAF^WT^, with approximately half of stage III and IV BRAF-mutant melanomas expressing high RHOJ levels. Treatment with FRAX597 suppresses the progression of RHOJ-expressing melanoma cells by preventing BAD phosphorylation, thereby restoring apoptotic sensitivity *in vitro* [68]. Because RHOJ–PAK signalling also promotes cell migration and invasion through actin cytoskeleton remodelling, its inhibition may impair tumour survival and metastatic dissemination [70]. Recent studies extend the role of RHOJ in mediating therapeutic resistance beyond melanoma to non-melanoma skin cancers. Using a mouse model of cutaneous squamous cell carcinoma undergoing EMT, Debaugnies *et al.* show that chemotherapy resistance is associated with elevated RHOJ levels. Mechanistically, RHOJ promotes DNA damage tolerance by facilitating nuclear actin fibre formation and reactivation of dormant replication origins, enhancing DNA repair and tolerance to replicative stress. Inhibition of actin polymerisation reinstates treatment sensitivity (Figure 3) [71].

Together, these studies establish RHOJ as a central regulator of EMT-associated plasticity and genotoxic stress adaptation, enabling tumour cells to withstand chemotherapy. The convergence of these functions positions RHOJ-centred signalling networks as promising therapeutic vulnerabilities, further reinforced by emerging roles in tumour angiogenesis that extend beyond the scope of this review [72]. However, despite compelling mechanistic evidence, most RHOJ-driven resistance programmes have been defined in melanoma models. Broader validation across diverse tumour types and clinically relevant *in vivo* systems will be essential to determine the generalisability and translational potential of targeting RHOJ signalling.

## RHOA-mediated mechanotransduction and survival signalling in therapy resistance

**Figure 4:**
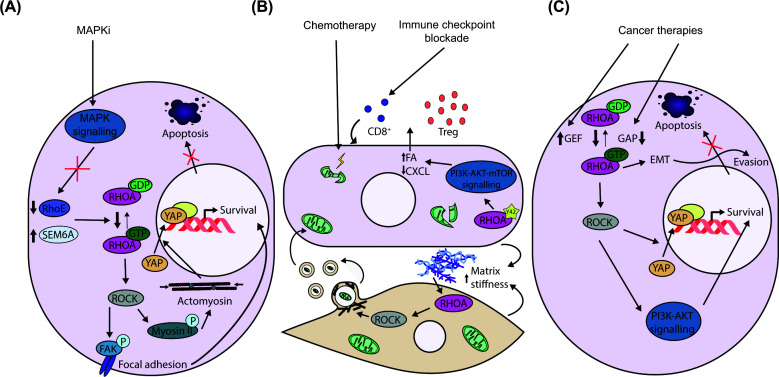
RHOA acts as a central node in cancer therapy resistance (**A**) Inhibition of MAPK signalling promotes resistance through enhanced actomyosin contractility and tumour survival. (**B**) RHOA modulates the tumour microenvironment to promote cancer cell survival and immunosuppression. (**C**) Cancer therapy triggers dysregulation of RHOA regulators, driving RHOA hyperactivation and downstream proliferation, EMT, and tumour survival.

RHOA-dependent signalling promotes therapeutic resistance across cancers by coupling actomyosin contractility and focal adhesion reinforcement to YAP-dependent survival programs, positioning RHOA as a central mediator of biomechanical adaptation to therapeutic stress. A paradigmatic example is observed in melanoma. Although combined BRAF and MEK inhibition with dabrafenib and trametinib is initially effective in BRAF^V600^-mutant melanoma, resistance frequently emerges after 12–24 months of continuous therapy. Transcriptomic analyses of patient-derived tumours before and after treatment reveal enrichment of extracellular matrix and focal adhesion gene signatures, consistent with enhanced mechanical signalling as a resistance driver. Mechanistically, MAPK inhibition down-regulates RHOE, an endogenous inhibitor of RHOA, relieving repression of RHOA activity and promoting activation of the RHOA–FAK–AKT axis (Figure 4A). Pharmacological inhibition of FAK using defactinib, combined with the RAF-MEK “clamp” avutometinib, overcomes resistance and improves efficacy in PDX [73,74]. ROCK-mediated myosin II activation has also been independently identified as a resistance mechanism to MAPK inhibition and immunotherapy in melanoma [75]. Together, these findings identify focal adhesion signalling as a clinically tractable vulnerability downstream of RHOA. RHOA-driven resistance is further reinforced by mechanotransduction pathways converging on YAP. The axon guidance molecule SEMA6A is preferentially expressed in BRAF-mutant melanoma cell lines and further induced upon BRAF and MEK inhibition. SEMA6A activates RHOA signalling and promotes YAP nuclear localisation, sustaining tumour cell survival under MAPK inhibition. Consistently, SEMA6A depletion restores sensitivity to BRAF and MEK inhibitors, implicating a RHOA-YAP axis in adaptive resistance (Figure 4A) [76].

## RHOA and tumour microenvironment-mediated resistance

Beyond tumour-autonomous mechanisms, RHOA also promotes resistance through tumour microenvironment interactions, where mechanical and metabolic cross-talk reinforce drug tolerance. In gastric cancer, oxaliplatin resistance is associated with increased mitochondrial content. Co-culture with mesenchymal cells plus oxaliplatin induces extracellular matrix remodelling, increasing matrix deposition and fibrosis. Under these conditions, mesenchymal stem cells release extracellular vesicles containing mitochondria, enabling mitochondrial transfer to cancer cells and promoting resistance (Figure 4B). Inhibition of RHOA disrupts vesicle release from mesenchymal stem cells, reducing microenvironment-driven resistance. Consistently, mitochondrial transfer is also blocked by the ROCK inhibitor Y-27632, which restores chemotherapy sensitivity (Table 1) [77]. In a complementary mechanism, the CX3CL1 chemokine is overexpressed in the gastric cancer microenvironment, and activation of the CX3CL1/CX3CR1 axis is associated with paclitaxel resistance. Pharmacological RHOA inhibition *in vivo* using the C3 transferase inhibitor CT04 reduces CX3CR1-associated paclitaxel resistance, implicating RHOA as a mediator of microenvironment-driven drug tolerance [78].

RHOA signalling has also been implicated in resistance to EGFR tyrosine kinase inhibitors, including osimertinib, in non-small cell lung cancer. In resistant tumours, expression of the lysosomal membrane protein *GLMP* is markedly up-regulated during treatment, correlating with advanced disease and poor prognosis. GLMP depletion in resistant cell lines restores drug sensitivity both *in vitro* and in CDX. Mechanistically, GLMP promotes autophagy and EMT through a RHOA-dependent pathway, whereas GLMP loss enhances RHOA ubiquitination and reduces RHOA protein levels. Consistently, RHOA depletion restores osimertinib sensitivity even with GLMP overexpression [79]. Beyond primary tumours, RHOA-mediated survival signalling is further amplified during brain metastatic outgrowth of EGFR-mutant lung cancer, where exposure to the mechanically and metabolically distinct brain microenvironment induces adaptive increases in RHOA activity. This microenvironment-driven rewiring promotes drug tolerance, and RHOA knockdown resensitises brain metastatic cells to osimertinib [80].

RHOA-mediated signalling is also linked to resistance to immune checkpoint blockade in gastric cancer. Tumours harbouring the RHOA^Y42^ mutation activate the PI3K-AKT-mTOR pathway, reducing chemokine production required for effector T-cell recruitment while enhancing fatty-acid synthesis that supports regulatory T-cell fitness. This metabolic reprogramming establishes an immunosuppressive niche favouring resistance to PD-1 blockade (Figure 4B). Consistently, RHOA mutations correlate with reduced immunotherapy responsiveness, and PI3K inhibition restores PD-1 sensitivity in RHOA^Y42^-mutant CDX [81].

## Dysregulated RhoGAP and RhoGEF control of RHOA in drug resistance

RHOA hyperactivation driven by RhoGAP loss or increased RhoGEF activity represents a common resistance mechanism. A genome-wide CRISPR screen identified ARHGAP35 as a determinant of sensitivity to regorafenib in liver cancer cells. ARHGAP35 loss sustains RHOA activation, promotes EMT, and suppresses apoptosis, whereas ARHGAP35 re-expression or RHOA inhibition with rhosin restores regorafenib sensitivity (Figure 4C) [82]. Elevated *RHOA* expression correlates with poor survival and reduced regorafenib efficacy in hepatocellular carcinoma. Genetic or pharmacological RHOA inhibition, using RNAi, Rhosin, or ARHGEF12 (LARG) inhibition with Y16, sensitises liver cancer cells to regorafenib, in part by reducing nuclear YAP activity and cancer stem cell properties (Table 1) [83]. In glioblastoma, ARHGEF12 expression correlates with tumour grade, and its depletion enhances temozolomide sensitivity in cell lines [84]. In bladder cancer cells, ARHGEF12 drives cisplatin resistance through RHOA-dependent PI3K-AKT activation, and ARHGEF12 depletion restores drug sensitivity, an effect phenocopied by ROCK inhibition with Y-27632 (Table 1 and Figure 4C) [85].

Dysregulated RHOA signalling is also observed in glucocorticoid-resistant leukemic cells, where overexpression of the long noncoding RNA *HOTAIRM1* represses *ARHGAP18*, enhancing RHOA–ROCK signalling and steroid resistance. This phenotype is reversed by ROCK1 inhibition with RKI-1447 (Table 1) [86]. RHOA also contributes to resistance to androgen deprivation therapy (ADT) in prostate cancer. In ADT-resistant cells, increased RACGAP1 activity promotes a switch from RAC- to RHOA-dependent signalling, elevating nuclear YAP and activating transcriptional programs that drive tumour growth and metastasis. Pharmacological inhibition of RHOA with simvastatin, which blocks RHOA geranylgeranylation, suppresses progression of androgen-resistant prostate cancer CDX models, while elevated nuclear YAP and RACGAP1 expression correlate with poorer patient survival [87,88].

Collectively, these findings position RHOA as a convergent node in therapy resistance, integrating biomechanical signalling, microenvironmental cross-talk, and metabolic rewiring to sustain tumour survival under therapeutic stress. Dysregulated control of RHOA by RhoGAP and RhoGEF proteins further amplifies this adaptive signalling, reinforcing the therapeutic potential of targeting RHOA-dependent pathways to overcome drug resistance. Future studies should focus on developing selective RHOA-pathway inhibitors and identifying predictive biomarkers to translate these mechanistic insights into clinically actionable strategies.

## Rho GTPases as therapeutic targets: are we there yet?

Mounting evidence implicates Rho GTPase-mediated signalling networks in therapeutic resistance, making them attractive yet challenging drug targets. Current strategies fall into three main categories: direct Rho GTPase inhibitors, inhibitors of GEF–GTPase interactions, and inhibitors of downstream effectors (Table 1).

Direct targeting of Rho GTPases has long been hindered by their compact structure, high-affinity GDP/GTP-binding site, and high intracellular GTP concentration. Structural studies have nonetheless identified the switch I and switch II regions as potential druggable surfaces, as conformational changes in these regions regulate interactions with GEFs, GAPs, and downstream effectors [3,89]. Current direct inhibitors mostly target switch II, which undergoes larger conformational rearrangements during nucleotide exchange and controls transitions between active and inactive states [90]. Inspired by the KRAS^G12C^ inhibitors, these compounds bind transient hydrophobic pockets exposed during conformational cycling, stabilising inactive states by disrupting nucleotide binding and interfering with GEF or effector engagement [91]. Morstein *et al.* demonstrated that inhibitors optimised for KRAS^G12C^ were also effective at targeting Rho GTPases upon the introduction of a matching G12C mutation, highlighting both the feasibility of this strategy and the need to develop inhibitors tailored to the switch II pocket of Rho GTPases [92]. However, high sequence and structural conservation among RAC, CDC42, and related family members remains a major obstacle, as off-target inhibition frequently leads to dose-limiting toxicity [3,93]. Ongoing structural analyses and molecular dynamics simulations aim to identify divergent residues, optimise binding kinetics, and refine inhibitor scaffolds to improve efficiency and selectivity [94].

Targeting GEF-GTPase interactions offers a conceptually appealing alternative, as GEFs integrate signals from oncogenic receptors and are often dysregulated in cancer [95,96]. In principle, GEF inhibition could enable pathway-selective modulation of Rho signalling. In practice, however, limited *in vivo* efficacy and extensive functional redundancy among GEFs have thus far constrained the therapeutic impact of this approach [97–99].

Among current strategies, inhibition of downstream effectors appears the most practical therapeutic strategy. Effectors such as ROCK and PAK possess druggable ATP-binding pockets, allowing selective interference with Rho-dependent outputs without directly inhibiting Rho GTPases. ROCK inhibitors slow cell migration, proliferation, and survival in multiple cancers without directly targeting RHOA, whereas PAK inhibition dampens major oncogenic outputs downstream of RAC1 and CDC42 [100,101]. Nonetheless, sustained effector inhibition can trigger compensatory signalling; for PAKs, this often involves activation of parallel effectors that affect cytoskeletal organisation, while ROCK blockade has been associated with increased survival of cancer stem cells, dormant tumour cells, and circulating tumour cells. Microenvironmental cues may further modulate these responses, occasionally enhancing chemoresistance or metastatic behaviour [102–104]. Moreover, high doses are often required due to competition with intracellular ATP, increasing off-target toxicity and unwanted effects on normal tissues [105–108].

Although several Rho pathway inhibitors show promise in preclinical models, few have advanced to clinical trials. Dose-limiting toxicity and suboptimal pharmacokinetics remain major barriers, underscoring the need for improved structure-guided optimisation and rational combination strategies to fully exploit Rho GTPase signalling as a therapeutic vulnerability.

## Perspectives

- Therapeutic resistance is a major driver of cancer mortality. By controlling EMT, cellular plasticity, and metastasis, Rho GTPases are key mediators of adaptive resistance and attractive therapeutic targets.
- Despite a strong biological rationale, Rho GTPases remain difficult to target directly. Structural constraints, high intracellular GTP levels, and limited *in vivo* validation have hindered drug development, and no FDA-approved therapies currently target Rho GTPases themselves.
- Because direct inhibition of RHO GTPases remains challenging, future strategies will likely emphasise indirect targeting while continuing to optimise existing inhibitors. Targeting downstream effectors such as ROCK and PAK, combined with structure-guided design and rational combination therapies, offers a promising path to overcome resistance.

## Open Access

Open access for this article is provided via our Subscribe to Open programme, made possible through the support of our subscribing institutions. Learn more here: https://portlandpress.com/pages/open_access_options_and_prices#conditional.

## Abbreviations

5-FU: 5-fluorouracil
ADT: androgen deprivation therapy
AR: androgen receptor
CDX: cell-line-derived xenografts
ER: estrogen receptor
GEFs: guanine nucleotide exchange factors
GAPs: GTPase-activating proteins
NOX: NADPH oxidases
PDX: patient-derived xenograft
